# Kitaev exchange and field-induced quantum spin-liquid states in honeycomb *α*-RuCl_3_

**DOI:** 10.1038/srep37925

**Published:** 2016-11-30

**Authors:** Ravi Yadav, Nikolay A. Bogdanov, Vamshi M. Katukuri, Satoshi Nishimoto, Jeroen van den Brink, Liviu Hozoi

**Affiliations:** 1 Institute for Theoretical Solid State Physics, IFW Dresden, Helmholtzstrasse 20, 01069 Dresden, Germany; 2 Department of Physics, Technical University Dresden, Helmholtzstrasse 10, 01069 Dresden, Germany; 3 Department of Physics, Harvard University, Cambridge, Massachusetts 02138, USA

## Abstract

Large anisotropic exchange in 5*d* and 4*d* oxides and halides open the door to new types of magnetic ground states and excitations, inconceivable a decade ago. A prominent case is the Kitaev spin liquid, host of remarkable properties such as protection of quantum information and the emergence of Majorana fermions. Here we discuss the promise for spin-liquid behavior in the 4*d*^5^ honeycomb halide *α*-RuCl_3_. From advanced electronic-structure calculations, we find that the Kitaev interaction is ferromagnetic, as in 5*d*^5^ iridium honeycomb oxides, and indeed defines the largest superexchange energy scale. A ferromagnetic Kitaev coupling is also supported by a detailed analysis of the field-dependent magnetization. Using exact diagonalization and density-matrix renormalization group techniques for extended Kitaev-Heisenberg spin Hamiltonians, we find indications for a transition from zigzag order to a gapped spin liquid when applying magnetic field. Our results offer a unified picture on recent magnetic and spectroscopic measurements on this material and open new perspectives on the prospect of realizing quantum spin liquids in *d*^5^ halides and oxides in general.

Quantum spin liquids (SL’s) are states of matter that cannot be described by the broken symmetries associated with conventional magnetic ground states^1^. Whereas there is a rich variety of mathematical models that exhibit SL behavior, finding materials in which a quantum SL state is realized is an intensely pursued goal in present day experimental condensed-matter physics^2^,^3^,^4^. Of particular interest is the Kitaev Hamiltonian on the honeycomb lattice^5^, which is a mathematically well-understood two-dimensional model exhibiting various topological SL states. Its remarkable properties include protection of quantum information and the emergence of Majorana fermions^5^,^6^.

The search to realize the Kitaev model of effectively spin-1/2 particles on the honeycomb lattice was centered until recently mainly on honeycomb iridate materials^7^,^8^ of the type *A*_2_IrO_3_, where *A* is either Na or Li. In these systems though long-range magnetic order develops at low temperatures, for all known different crystallographic phases^9^,^10^,^11^,^12^,^13^. The SL regime is most likely preempted in the iridates by the presence of significant residual Heisenberg-type *J* couplings, by longer-range spin interactions, or by having crystallographically distinct Ir-Ir bonds with dominant *J*’s on some of those, if not a combination of these factors^14^,^15^,^16^,^17^.

Also of interest in this context is ruthenium trichloride, RuCl_3_, in its honeycomb (*α*) crystalline phase^18^,^19^,^20^,^21^,^22^,^23^,^24^,^25^,^26^. Very recent Raman and neutron scattering measurements suggest that the 4*d*^5^ halide honeycomb system is closer to the Kitaev limit^22^,^23^. But also this material orders antiferromagnetically at low temperatures, as the 5*d*^5^ iridium oxides do, and precisely how close to the idealized Kitaev model *α*-RuCl_3_ is, remains a question to be clarified.

Here we present results of combined quantum chemistry electronic-structure computations and exact-diagonalization (ED) calculations for extended Kitaev-Heisenberg spin Hamiltonians using as starting point for the ED study the magnetic couplings derived at the quantum chemistry level. Our results for the Ru^3+^ 4*d*-shell electronic structure show sizable trigonal splitting of the 4*d t*_2*g*_ levels and therefore a spin-orbit ground state that significantly deviates from the *j*_eff_ = 1/2 picture^7^. The trigonally distorted environment further gives rise to strong anisotropy of the computed *g* factors, consistent with experimental observations^20^,^27^. Calculating the magnetic interactions between two adjacent 1/2-pseudospins, we find that the nearest-neighbor (NN) Kitaev exchange *K* is ferromagnetic (FM), in any of the *α*-RuCl_3_ crystalline structures reported so far. It is however significantly weaker than in 5*d*^5^ Ir oxides and even than in 4*d*^5^ Li_2_RhO_3_, which points at a rather different balance between the various superexchange processes in the halide and in the oxides.

The resulting magnetic phase diagram that we compute as function of longer-range second- and third-neighbor magnetic couplings is very rich, due to the comparable size of the various residual interactions. While a SL state does show up in this phase diagram, it arises in a setting different from Kitaev’s original SL regime, as it emerges from an interplay of Kitaev physics and geometrically frustrated magnetism. We additionally find that applying an external magnetic field whilst the system is in the long-range ordered zigzag ground state can induce a phase transition into a quantum SL. In order to make direct contact with experimental observations, we calculate by ED the field-dependent magnetization in the presence of longer-range magnetic interactions and compare that to the measurements. This comparison makes clear that the ED and experimental data can only be matched when *J* is small and antiferromagnetic (AF) and *K* significantly stronger and FM, in accordance with the results from the *ab initio* quantum chemistry calculations.

The magnitude of our computed |*K*| compares well with recent estimates based on neutron scattering^23^ and Raman^22^ data. However, our finding that *K* is FM brings into question the interpretation of the neutron scattering experiments in terms of a pure Kitaev-Heisenberg model with AF *K* but without longer-range magnetic couplings which we find to be essential for an understanding of the magnetic properties of *α*-RuCl_3_.

## Spin-orbit ground state and excitations

We start our discussion with the analysis of the Ru^3+^ 4*d*-shell electronic structure. As in the 5*d*^5^ iridates, the magnetic moments in *α*-RuCl_3_ are related to the one hole in the transition-metal *t*_2*g*_ subshell, described by the effective *L* = 1 angular-momentum and *S* = 1/2 spin quantum numbers. Even if the spin-orbit coupling (SOC) for 4*d* electrons is weaker than in the Ir 5*d* orbitals, it still splits the 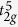 states into a *j*_eff_ = 1/2 sector, where the hole resides, and a *j*_eff_ = 3/2 manifold that is filled. But for noncubic environment, these *j*_eff_ = 1/2 and *j*_eff_ = 3/2 components may display some degree of admixture.

Three different crystallographic structures^28^,^29^,^30^ have been reported for *α*-RuCl_3_, each of those displaying finite amount of trigonal compression of the Cl_6_ octahedra. To shed light on the nature of the 1/2-pseudospin in *α*-RuCl_3_ we first discuss in this section results of *ab initio* many-body calculations at the complete-active-space self-consistent-field (CASSCF) and multireference configuration-interaction (MRCI) levels of theory^31^ for embedded atomic clusters having one RuCl_6_ octahedron as reference unit.

As shown in Table 1, the degeneracy of the Ru *t*_2*g*_ levels is completely removed, with CASSCF splittings of 69 and 72 meV when using the RuCl_3_ *C*2/*m* structure determined by Cao *et al*.^28^, a minimal active orbital space of only three 4*d* orbitals and no SOC. A “trigonal” orbital basis is used in Table 1 to express the 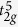 wave functions^27^, in contrast to the Cartesian orbital basis employed for the Rh^4+^ 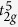 states in ref. 32, better suited for Li_2_RhO_3_ due to additional distortions of the ligand cages giving rise in the rhodate to one set of longer ligand-metal-ligand links with an angle of nearly 180°.

The corrections brought by the MRCI treatment are tiny, smaller than in the 4*d* oxide^32^ Li_2_RhO_3_ due to less metal-*d* – ligand-*p* covalency in the halide. The smaller effective ionic charge at the ligand sites in the halide — Cl^−^ in RuCl_3_ vs O^2−^ in Li_2_RhO_3_, in a fully ionic picture — further makes that the transition-metal *t*_2*g*_ − *e*_*g*_ ligand-field splitting is substantially reduced in RuCl_3_: by MRCI calculations without SOC but with all five Ru 4*d* orbitals active in the reference CASSCF, we see that the lowest 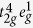 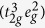 states are at only 1.3 (1.5) eV above the low-lying 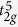 component (see Table 2). Interestingly, for the “older” *P*3_1_12 crystal structure proposed in ref. 29, we find that the 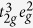 sextet lies even below the lowest 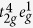 states, see Methods. The smaller effective ligand charge might also be the cause for the smaller *t*_2*g*_-shell splittings in the halide: ≈70 meV in RuCl_3_ (see caption of Tables 1 and 2) vs ≈90 meV in Li_2_RhO_3_^32^ at the MRCI level, in spite of having similar degree of trigonal compression in these two materials.

With regard to the split *j*_eff_ = 3/2-like states that we compute at 195 and 234 meV by MRCI + SOC calculations involving all three 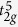, 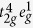 and 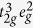 configurations in the spin-orbit treatment (see Table 2), clear excitations have been measured in that energy range in Raman scattering experiments with “crossed” polarization geometries^22^,^26^ and also in the optical response of *α*-RuCl_3_^18^,^26^. The peak observed at 140–150 meV by Raman scattering^26^, in particular, may find correspondence in the lowest *j*_eff_ = 3/2-like component that we compute at 195 meV. It is interesting that in Sr_2_IrO_4_ the situation seems reversed as there the Raman selection rules appear to favor the higher-energy split-off 3/2 states^33^, which are however shifted to somewhat lower energy as compared to resonant inelastic x-ray scattering (RIXS)^34^. One should note however that in Sr_2_IrO_4_ the crystal-field physics is rather subtle, as the local tetragonal distortion giving rise to elongated apical bonds is counteracted by interlayer cation charge imbalance effects^35^.

The rather broad feature at 310 meV in the imaginary part of the dielectric function has been assigned to Ru^3+^ *t*_2*g*_-to-*e*_*g*_ transitions^26^. Our *ab initio* data do not support this interpretation, since the lowest *t*_2*g*_ → *e*_*g*_ excitations are computed at ≈1.3 eV, but rather favor a picture in which the 310 meV peak corresponds to the upper 3/2-like component. The latter can become optically active through electron-phonon coupling. The rather large width of that excitation has been indeed attributed to electron-phonon interactions in ref. 26.

Comparison between our quantum chemistry results and the optical spectra^18^,^26^ further shows that the experimental features at 1.2 and 2 eV, assigned in ref. 26 to intersite *d*–*d* transitions, might very well imply *on*-*site* Ru 4*d*-shell excitations. In particular, we find spin-orbit states of essentially 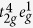 nature at 1.3–1.5 eV and of both 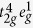 and 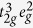 character at 1.7–2.2 eV relative energy, see Table 2. Experimentally the situation can be clarified by direct RIXS measurements on *α*-RuCl_3_, for instance at the Ru *M*_3_ edge.

We have also calculated the magnetic *g* factors in this framework. By spin-orbit MRCI calculations with all five Ru 4*d* orbitals in the reference CASSCF, we obtain for the *C*2/*m* structure of ref. 28 *g*_*ab*_ = 2.51 and *g*_*c*_ = 1.09, where the crystallographic *c* axis is perpendicular to the (*ab*) Ru honeycomb plane. On the experimental side, conflicting results are reported for the *g* factors: while Majumder *et al*.^19^ derive from magnetic susceptibility data that both *g*_*ab*_ and *g*_*c*_ are 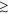
2, Kubota *et al*.^20^ estimate *g*_*ab*_ = 2.5 and *g*_*c*_ = 0.4. The latter *g*_*c*_ value implies a rather large *t*_2*g*_-shell splitting *δ*, with *δ*/*λ* > 0.75 (see the analysis in ref. 20). The quantum chemistry *g* factors are consistent with a ratio *δ*/*λ* ~ 0.5, i.e., *t*_2*g*_ splittings of ≈70 meV (see the data in Tables 1 and 2) for a 4*d* SOC in the range of 120–150 meV^24^,^27^,^36^. Electron spin resonance measurements of the *g* factors might provide more detailed experimental information that can be directly compared to our calculations.

## Intersite exchange for *j* **≈** 1/2 moments

NN exchange coupling constants were derived from MRCI + SOC calculations for embedded fragments having two edge-sharing RuCl_6_ octahedra in the active region. As described in earlier work^16^,^17^,^32^,^35^, the *ab initio* data for the lowest four spin-orbit states describing the magnetic spectrum of two NN octahedra is mapped in our scheme onto an effective spin Hamiltonian including both isotropic Heisenberg exchange and symmetric anisotropic interactions. Yet the spin-orbit calculations, CASSCF or MRCI, incorporate all nine triplet and nine singlet low-energy states of predominant 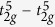 character. As in earlier studies^16^,^17^,^32^,^35^, we account in the MRCI treatment for all single and double excitations out of the valence *d*-metal *t*_2*g*_ and bridging-ligand *p* shells.

For on-site Kramers-doublet states, the effective spin Hamiltonian for a pair of NN ions at sites *i* and *j* reads


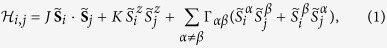


where 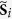 and 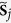 are 1/2-pseudospin operators, *J* is the isotropic Heisenberg interaction, *K* the Kitaev coupling and the Γ_*αβ*_ coefficients are off-diagonal elements of the symmetric anisotropic exchange matrix with *α, β* ∈ {*x, y, z*}. Since the point-group symmetry of the Ru–Ru links is *C*_2*h*_ in the *C*/2*m* unit cell, the antisymmetric Dzyaloshinskii-Moriya exchange is 0. Also, Γ_*zx*_ = −Γ_*yz*_ for *C*_2*h*_ bond symmetry. A local (Kitaev) reference frame is used here, such that for each Ru-Ru link (see Table 3) the *z* axis is perpendicular to the Ru_2_Cl_2_ plaquette (as also employed in refs 16, 17 and 32). Details of the mapping procedure, *ab initio* data to effective spin Hamiltonian, are described in ref. 35 and Methods.

From the quantum chemistry calculations, we obtain a FM Kitaev coupling *K*, for all three crystalline structures reported in the literature (see Table 3). Its strength is reduced as compared to the 4*d*^5^ honeycomb oxide Li_2_RhO_3_^32^, with a maximum absolute value of 5.6 meV in the *C*2/*m* structure proposed by Cao *et al*.^28^. We shall discuss and compare our finding of a FM Kitaev coupling to other theoretical and experimental findings in the next section. For the structure of Cao *et al*.^28^ the bond lengths and angles are very similar for the two types of pairs of NN octahedra. As a result, we find identical effective interactions up to the first digit. This is the reason we provide in Table 3 only one set of couplings for that particular crystal structure. Anisotropic interactions of similar size, i.e., both *K* and the off-diagonal couplings Γ_*αβ*_, are computed for the *C*2/*m* configuration of ref. 30, characterized by bond lengths and bond angles rather close to the values derived by Cao *et al*.^28^. The Heisenberg *J*, on the other hand, changes sign with decreasing Ru-Cl-Ru flexure but for the bond angles reported in refs 28, 29, 30 and explicitly given in Table 3 remains in absolute value significantly smaller than *K*.

**Figure 1:**
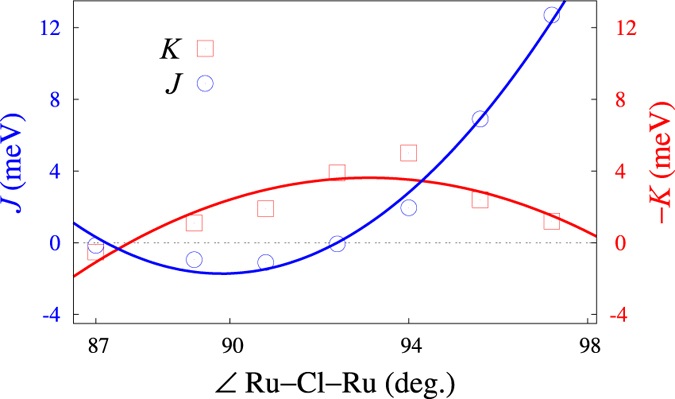
Variation of the NN Heisenberg and Kitaev couplings with the Ru-Cl-Ru angle in model *C*2/*m*-type structures. Results of spin-orbit MRCI calculations. The NN Ru-Ru distance is set to 3.44 Å and the Ru-Cl bond lengths are for each angle all the same. The variation of the Ru-Cl-Ru angle is the result of gradual trigonal compression. Curves are drawn just as a guide for the eye.

The trends we find with changing the Ru-Cl-Ru bond angle, apparent from Table 3, and earlier results for the dependence of *K* and *J* on bond angles in oxide honeycomb compounds^17^,^32^ motivate a more detailed investigation over a broader range of Ru-Cl-Ru flexure. The outcome of these additional calculations is illustrated in Fig. 1. We fixed in these calculations the Ru-Ru distance to 3.44 Å and varied the Ru-Cl-Ru angle by changing the amount of trigonal compression for each of the two NN RuCl_6_ octahedra. In contrast to the oxides, where |*K*| values in the range of 15–30 meV are computed for large angles of 98–100°, the Kitaev coupling is never as strong in RuCl_3_. |*K*| shows a maximum of only ≈5 meV at 94° in Fig. 1 and its angle dependence is far from the nearly linear behavior in 4*d*^5^ and 5*d*^5^ oxides^17^,^32^.

The Heisenberg *J*, on the other hand, displays a steep upsurge with increasing angle, more pronounced as compared to the honeycomb oxides. In other words, for large angles *J* dominates in RuCl_3_, in contrast to the results found in 4*d*^5^ and 5*d*^5^ honeycomb oxides in the absence of bridging-ligand displacements parallel to the metal-metal axis^17^,^32^. These notable differences between the halide and the oxides suggest a somewhat different balance between the various superexchange processes in the two types of systems.

## Magnetic phase diagram

To assess the consistency of our set of *ab initio* NN effective couplings with experimental observations, we carried out ED calculations for the 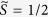 honeycomb model described by (1) but including additionally the effect of second- and third-neighbor *J*_2_ and *J*_3_ isotropic exchange. Anisotropic longer-range interactions are however neglected since recent phenomenological investigations conclude those are not sizable^37^. We first considered the case without external magnetic field, *H* = 0, and clusters of 24 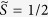 sites with periodic boundary conditions (PBC’s). The static spin-structure factor 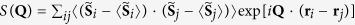 was calculated as a function of variable *J*_2_ and *J*_3_ parameters while fixing the NN couplings to the MRCI results computed for the crystalline structure of ref. 28 and listed in Table 3.

**Figure 2:**
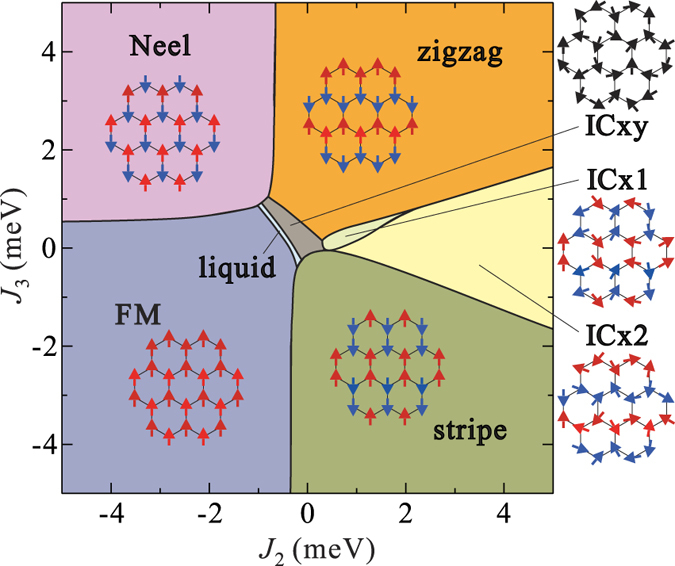
Phase diagram for the effective spin model of (1) supplemented by second- and third-neighbor Heisenberg couplings *J*_2_ and *J*_3_. MRCI NN interactions as listed on first entry in Table 3 were used: *J* = 1.2, *K* = −5.6, Γ_*xy*_ = −1.2, Γ_*zx*_ = −Γ_*yz*_ = −0.7 (meV). Schematic spin configurations for each particular phase are also shown. No external field is applied in this set of calculations (*H* = 0).

For a given set of *J*_2_ and *J*_3_ values, the dominant order is determined according to the propagation vector **Q** = **Q**_*max*_ providing a maximum value of *S*(**Q**). As shown in Fig. 2, the phase diagram contains seven different phases: four commensurate phases (FM, Néel, zigzag, stripy), three with incommensurate (IC) order (labelled as ICx1, ICx2, ICxy) and a SL phase. The ICx1 and ICx2 configurations have the same periodicities along the *b* direction as the stripy and zigzag states, respectively, and display IC wave numbers along *a*. The ICxy phase has IC propagation vectors along both *a* and *b*. The variety of IC phases in the computed phase diagram is related to the comparable strength of the NN *J* and the off-diagonal NN couplings Γ_*αβ*_. For example, the system is in the ICxy state for *J*_2_ = *J*_3_ = 0. From the experimental observations, the low-temperature magnetic structure of *α*-RuCl_3_ is *ab*-plane zigzag AF order^21^,^28^,^30^. We find indeed that the zigzag state is stabilized in a wide range of AF *J*_2_ and *J*_3_ values in our phase diagram.

**Figure 3:**
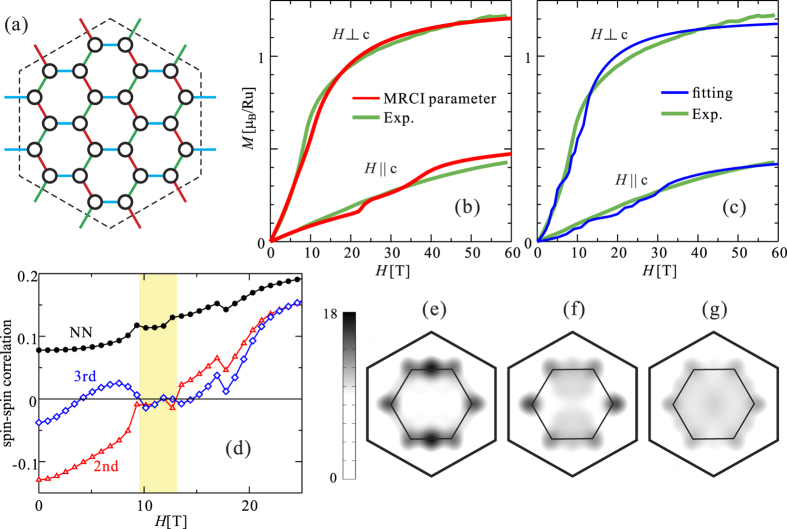
Magnetization curves and spin-structure factors for *α*-RuCl_3_. (**a**) 24-site cluster with PBC’s used in the ED calculations. (**b**) Experiment^30^ vs ED plots using MRCI *g* factors and NN couplings plus *J*_2_ = *J*_3_ = 0.25 meV. (**c**) ED magnetization curves with *J* = 1.0, *K* = −5.0, *J*_2_ = *J*_3_ = 0.3, *g*_*ab*_ = 2.4, *g*_*c*_ = 0.95 and vanishing off-diagonal NN couplings. (**d**) Field dependence of the NN, second- and third-neighbor spin-spin correlation functions 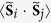 extrapolated to the thermodynamic limit, using MRCI *g* factors and NN couplings, *J*_2_ = *J*_3_ = 0.25 meV and field along the *c* axis. Static spin-structure factors *S*(**Q**) are shown for (**e**) *H* = 0, (**f**) *H* = 9.5, (**g**) *H* = 10.5 T.

To estimate the strength of *J*_2_ and *J*_3_ in *α*-RuCl_3_, we performed a fitting of the experimental magnetization curves^30^ by ED calculations. The PBC cluster we used is shown in Fig. 3(a). We find that different signs for *J* and *K* determine qualitatively different shapes for the magnetization curves. In particular, *J* > 0 and *K* < 0 values are required to reproduce the overall pattern of the measured magnetization, which exhibits a very slow saturation with increasing external field (see Methods). Additionally, AF values for *J*_2_ and *J*_3_ significantly shift the saturation to higher field and therefore these longer-range couplings must be small (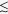
1 meV, see Methods) to reproduce the experiment. The observed magnetization curves are set side by side to ED results in Fig. 3(b), for both 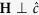 and 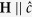. We used MRCI *g* factors (*g*_*ab*_ = 2.51, *g*_*c*_ = 1.09) and MRCI NN interactions (see Table 3) and set *J*_2_ = *J*_3_ = 0.25 meV in Fig. 3(b). It is seen that the overall shapes of the experimental curves are well reproduced in these ED calculations. For comparison, additional ED results are provided in Fig. 3(c) with *J* = 1.0, *K* = −5.0, *J*_2_ = *J*_3_ = 0.3, *g*_*ab*_ = 2.4, *g*_*c*_ = 0.95 and vanishing off-diagonal NN couplings. It is difficult to extract information on the latter by using ED fits to the experimental data because the magnetization is not very sensitive to the strength of these off-diagonal NN exchange interactions. The magnetization is very sensitive, on the other hand, to the *g* factors — its strong directional dependence mainly comes from the strongly anisotropic *g* factors.

Most interestingly, a level crossing between the lowest two states is seen around *H* = 10 T for **H** ‖ [001] in all periodic clusters we considered. To better understand the nature of the changes at this level crossing, we analyzed the spin-spin correlation functions 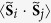. Results in the thermodynamic limit are presented in Fig. 3(d), while a detailed finite-size scaling analysis and further discussion on the spin correlations are provided in Methods. Below *H* ≈ 10 T the zigzag AF correlations are dominant; only the NN spin-spin correlations being large for fields of 10–13 T (magnetization *M*/*M*_s_ ~ 0.25 − 0.45) is indicative of a Kitaev-like SL regime. The level crossing can be therefore associated to a transition between AF zigzag order and a SL. Static spin-structure factors for *H* = 0, 9.5 and 10.5 T are plotted in Fig. 3(e–g). A featureless static spin-structure factor is obtained for *H* > 10 T, consistent with the spin-spin correlations shown in Fig. 3(d). In other words, we argue that the zigzag AF order is gradually weakened with increasing *H*, destroyed at *H* = 10 T and instead a SL ground state occurs for *H* > 10 T.

The MRCI calculations indicate |*K*|/*J* ratios in a range of 3 to 5 for the *C*/2*m* structures (see Table 3) while a commonly used criterion^8^ for identifying the Kitaev SL is having |*K*|/*J* > 7.8, so that the further frustration of magnetic interactions is relevant as well. One simple way to rationalize these findings is that an external field effectively weakens the effect of the AF NN *J* due to partial spin polarization and consequently |*K*|/*J* is effectively enhanced. Another way of qualitatively appreciating this point is that when one looks at the *J*_2_–*J*_3_ phase diagram in Fig. 2, the main features of which are very similar to those^16^ found for Na_2_IrO_3_, a trajectory in the phase diagram from zigzag order (the low field state) to a saturated ferromagnet (the very high field state) is likely to pass through the SL phase. It is interesting that such a field-induced SL state due to frustration has been also predicted recently for the *S* = 1/2 AF kagomé lattice^38^.

In the Kitaev limit, it is confirmed by earlier density-matrix renormalization group (DMRG) calculations that topological phases can survive up to *M*/*M*_s_ ≈ 0.5^39^, a critical value in agreement with our upper bound of the SL phase. It may be that due to the longer-range *J*_2_ and *J*_3_ couplings the topological phase in the low-field regime of the Kitaev limit^39^ is replaced by the zigzag ground state in our model.

**Figure 4:**
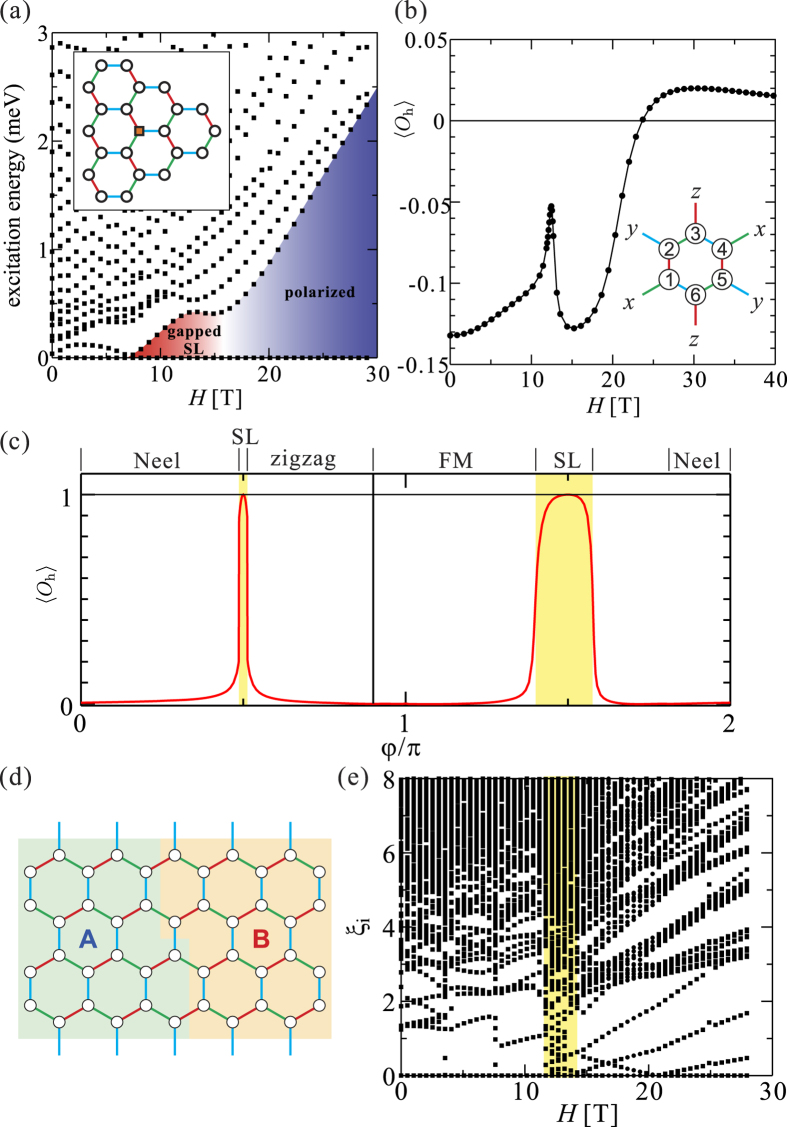
Excitation spectrum, plaquette operator and entanglement spectrum for *α*-RuCl_3_. (**a**) Energy spectrum *E*_*ν*_ − *E*_0_ as a function of *H* (‖[001]) using the MRCI *g* factors and NN couplings plus *J*_2_ = *J*_3_ = 0.25 meV. Inset: 22-site cluster with OBC’s used in the ED calculations; the square indicates a target site to apply the spin-flip operator. (**b**) Expectation value of *O*_h_ as a function of *H* (‖[001]). Inset: labeling for the (hexagonal) plaquette operator *O*_h_. (**c**) 〈*O*_h_〉 vs *φ* plot for the plain NN Kitaev-Heisenberg model. (**d**) 44-site cylindrical cluster used in the DMRG calculation of entanglement spectra, with PBC’s for the vertical direction. (**e**) Resulting entanglement spectrum (*H* ‖ [001]); the off-diagonal NN couplings are set to 0 in the DMRG computations, *J*_2_ = *J*_3_ = 0.25.

We also analyzed the gap Δ in the SL state. Due to discrete effects in clusters with PBC’s 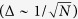, this analysis was performed using a setup with open boundary conditions (OBC’s). To remove artifacts related to individual motions around the open edges, we calculated the excitation spectrum for a spin flip at a site in the central region of the cluster,


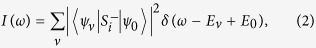


where 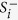 is the spin-flip operator at site *i* and |*ψ*_*ν*_〉 and *E*_*ν*_ are eigenstates and eigenvalues of the system, respectively (*ν* = 0 corresponds to the ground state). The position of site *i* and the computed spectrum *E*_*ν*_ − *E*_0_ are shown in Fig. 4(a). Obviously, a gap linear in *H* (Δ ∝ *H* − *H*_s_) in high fields (*H* > *H*_s_ ≈ 15 T) indicates a fully polarized FM state. But a sizable gap opens as well for fields in the range of 8–15 T, in spite of having no long-range magnetic order. This is another result that indicates a gapped SL state.

Furthermore, to check the topological properties of the gapped SL state, we considered the hexagonal plaquette operator^5^


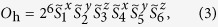


where the labeling of links and sites is illustrated in the inset of Fig. 4(b). The expectation value of *O*_h_ was calculated using the 24-site PBC cluster. Results for *α*-RuCl_3_ are provided as a function of *H* in Fig. 4(b). For comparison, a plot of 〈*O*_h_〉 for the plain Kitaev-Heisenberg model is shown in Fig. 4(c) (the definition of the Kitaev-Heisenberg Hamiltonian is given in Methods). In the Kitaev limit, the operator (3) commutes with the Hamiltonian and the expectation value 〈*O*_h_〉 is exactly ±1. On the other hand, it rapidly drops to 〈*O*_h_〉 ~ 0 when moving away from the Kitaev SL regime. The expectation value we compute for *α*-RuCl_3_ is 〈*O*_h_〉 ≈ −0.13 at *H* = 0. It monotonously decreases in absolute value with increasing *H* and displays a steep enhancement at *H* = 12.3 T. The absolute value of 〈*O*_h_〉 in the SL regime (fields of 12.3–16.1 T) is significantly lower than the limit 〈*O*_h_〉 = ±1 for the pure Kitaev model, pointing again to the important role of longer-range AF interactions. The second-neighbor couplings, in particular, frame a triangular AF Heisenberg net — a well-known playground for frustration-induced SL physics.

Finally, for insights into the topological properties of the system, we investigated by DMRG methods the field dependence of the entanglement spectrum (ES)^40^. Using Schmidt decomposition, the ground state can be expressed as


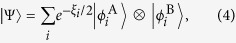


where the states 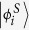 correspond to an orthonormal basis for the subsystem *S* (either A or B). We studied a cylindrical cluster with 44 sites whose subdomains A and B are sketched in Fig. 4(d). In our calculations, the ES {*ξ*_*i*_} is simply obtained as *ξ*_*i*_ = −log *λ*_*i*_, where {*λ*_*i*_} are the eigenvalues of the reduced density matrices after the bipartite splitting. The low-lying ES levels are plotted as function of magnetic field in Fig. 4(e). A relatively large “gap” is seen below *H* = 11.5, since the AF zigzag state is topologically trivial. With increasing *H*, a crossover is clearly seen around *H* = 11.5 T. Interestingly, there exist many (nearly) degenerate low-lying levels for fields in the interval 11.5–14 T. This is the window for which a SL ground state is suggested by the behaviour of other quantities and parameters discussed above. The low-lying levels are distributed in a rather broad range of the partition spin sectors: for example, at *H* = 13.2 T, *ξ*_1_ = 0 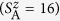, *ξ*_2_ = 0.0097 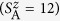, *ξ*_3_ = 0.1697 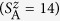, *ξ*_4_ = 0.4243 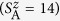, *ξ*_5_ = 0.4823 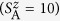, *ξ*_6_ = 0.5327 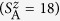, *ξ*_7_ = 0.7968 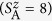 etc., where 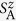 is the total *S*^*z*^ of subsystem A. This also supports the appearance of the SL state. For higher fields, the *ξ*_2_ − *ξ*_1_ gap increases linearly, reflecting the field-induced FM state.

## Discussion

Our finding of a FM Kitaev interaction can be first compared with the conclusions of other theoretical investigations. In fact, the analysis of effective superexchange models using hopping matrix elements and effective Hubbard-*U* interactions derived from density-functional (DF) electronic-structure calculations lead to contradictory results: an AF NN Kitaev coupling has been earlier predicted by Kim *et al*.^24^ and a FM *K* has been more recently found by Winter *et al*.^37^. Our result is qualitatively consistent with the latter. Relevant in this regard is further the trends we observe for the effective *K* by running spin-orbit calculations at different levels of approximation: restricted open-shell Hartree-Fock (ROHF), CASSCF and MRCI. The respective *K* values are 1.2, −2.5 and −5.6 meV, for the *C*2/*m* structure of ref. 28. It is seen that accounting for intersite *t*_2*g*_ − *t*_2*g*_ hopping by CASSCF changes the sign of *K* from AF to FM and that by additionally taking into account superexchange paths involving the bridging-ligand 3*p* and metal *e*_*g*_ levels by MRCI calculations with single and double excitations only pushes *K* more on the FM side. It is unlikely that additional excitations, “triple” etc., would change the sign of *K* back to the AF ROHF.

To make direct contact with experimental observations, one can compare the measured field-dependent magnetization with the theoretical results, as we did above, finding that only *J* > 0 and *K* < 0 are consistent with the measurements^30^. This however contradicts the interpretation of recent inelastic neutron scattering data on the magnetic excitation spectrum^23^, according to which *K* is very similar in magnitude to our finding but AF.

This point remains to be clarified but a possible explanation is related to modeling the experimental magnetic excitation spectra in the zigzag ordered state in terms of a pure Kitaev-Heisenberg Hamiltonian without longer-range couplings. In such a restricted model, zigzag order can *only* occur when *J* < 0 and *K* > 0, i. e., using the zigzag ordered ground state as input for the pure Kitaev-Heisenberg model fixes *K* > 0 from the beginning and a description of the magnetic excitations on top of this ground state in terms of linear spin-wave theory is necessarily confined to this boundary condition. We find however that *α*-RuCl_3_ is in a parameter regime where *without* longer-range, second-neighbor and third-neighbor, interactions the ordering pattern would be an incommensurate AF state (see Fig. 2) which is close to the stripe-like AF phase. This is the consequence of having *J* > 0 and *K* < 0. A weak AF third-neighbor exchange *J*_3_ is essential to stabilize the zigzag order that is experimentally observed — this zigzag ground state is driven by the geometric frustration induced by *J*_3_ and consistent with *K* being dominant and FM.

For an interpretation of the magnon features in the neutron spectrum ref. 23 employs linear spin-wave theory while for resolving the signatures of the fractionalized excitations — the actual fingerprint of the system being proximate to a Kitaev SL state — relies on a comparison to a Kitaev-only Hamiltonian. This should provide a full quantum description of the relevant physics on energy scales larger than weak interlayer magnetic couplings. The Kitaev point is particularly interesting because exact statements can be made^5^,^41^,^42^. In the honeycomb Kitaev model the excitations are exactly fractionalized into localized fluxes and delocalized Majorana modes. Its dynamic spin-structure factor, which determines the inelastic neutron scattering response, is dominated by a spin excitation creating two fluxes. As the fluxes are localized, the spin-structure factor is rather dispersionless and only a weak momentum dependence arises from screening of the fluxes by gapless Majorana modes^41^. The sign of *K* sets the sign for the dispersion of these Majorana modes that screen the fluxes^5^. The upshot is that the dynamic structure factor in the Kitaev model strongly depends on the magnitude of |*K*| (which sets the energy threshold for flux creation) but only very weakly on its sign — fits to the data with |*K*| and −|*K*| then provide very similar results.

## Conclusions

In sum, quantum chemistry calculations show that in *α*-RuCl_3_ there is sizable trigonal splitting of the Ru 4*d*^5^ levels. This results in splitting of the spin-orbit excitation energies, which can be accurately measured by e.g. resonant inelastic x-ray scattering, and in admixture of the *j*_eff_ = 1/2 and *j*_eff_ = 3/2 states. The resulting anisotropy of the magnetic *g* factors that we compute is consistent with experimental observations^20^.

The nearest-neighbor Heisenberg interaction *J* is found to be weak and antiferromagnetic in the *ab initio* computations while the Kitaev *K* is 3–5 times larger and ferromagnetic. Using these magnetic couplings as a basis for effective-model exact-diagonalization calculations of the magnetic phase diagram, we show that *J* > 0 and *K* < 0 values are required to reproduce the shape of the observed magnetization. The latter exhibits a very slow saturation with increasing the external field. As residual longer-range magnetic interactions would significantly shift the saturation to higher field, these couplings must be small. At the same time, however, we find the longer-range couplings are essential in producing the experimentally observed zigzag magnetic order in *α*-RuCl_3_.

We also determine by quantum chemistry calculations the dependence of the NN *K* and *J* interactions on the angle defined by two adjacent metal sites and a bridging ligand. Along with similar curves we have computed for the “213” honeycomb compounds^17^,^32^ — Na_2_IrO_3_, Li_2_IrO_3_ and Li_2_RhO_3_ — these results provide theoretical benchmarks for strain and pressure experiments on 4*d*^5^/5*d*^5^ honeycomb halides and oxides.

In our numerical investigations, a level crossing between the lowest two states is seen for field along the [001] direction around *H* = 10 T, i. e., a transition from AF zigzag order to a gapped spin-liquid state. We note that qualitatively similar features are also found for other field directions. Our calculations suggest that not only *α*-RuCl_3_ but also Na_2_IrO_3_ is a candidate material to observe such a transition, either at low-temperature ambient conditions or under external pressure.

## Methods

### Ru^3+^ 4*d*-shell electronic structure

*Ab initio* many-body quantum chemistry calculations were first carried out to establish the nature of the Ru^3+^ 4*d*^5^ ground state and lowest Ru 4*d*-shell excitations in RuCl_3_. An embedded cluster having as central region one [RuCl_6_]^3−^ octahedron was used. To describe the finite charge distribution in the immediate neighborhood, the three adjacent RuCl_6_ octahedra were also explicitly included in the quantum chemistry computations while the remaining part of the extended solid-state matrix was modeled as a finite array of point charges fitted to reproduce the ionic Madelung field in the cluster region^43^. Energy-consistent relativistic pseudopotentials were used for the central Ru ion, along with valence basis sets of quadruple-zeta quality augmented with two *f* polarization functions^44^. For the Cl ligands of the central RuCl_6_ octahedron, we employed all-electron valence triple-zeta basis sets^45^. For straightforward and transparent analysis of the on-site multiplet physics (see Table 2 in main text and Table 4 in this section), the adjacent Ru^3+^ sites were described as closed-shell Rh^3+^ 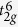 ions, using relativistic pseudopotentials and valence triple-zeta basis functions^44^. Ligands of these adjacent octahedra that are not shared with the central octahedron were modeled with all-electron minimal atomic-natural-orbital basis sets^46^. Results in excellent agreement with the experiment were found by using such a procedure in, e.g., Sr_2_IrO_4_^35^ and CaIrO_3_^47^.

All computations were performed with the Molpro quantum chemistry package^48^. To access the Ru on-site excitations, we used active spaces of either three (see Table 1 in main text) or five (Table 2 in main text and Table 4 in this section) orbitals in CASSCF. In the subsequent MRCI^49^,^50^, the Ru *t*_2*g*_ and Cl 3*p* electrons at the central octahedron were correlated. The Pipek-Mezey localization module^51^ available in Molpro was employed for separating the metal 4*d* and Cl 3*p* valence orbitals into different groups, i. e., centered at sites of either the central octahedron or of the adjacent octahedra. The spin-orbit treatment was carried out as described in ref. 52.

One important finding in our quantum chemistry investigation is that compared to the 4*d* and 5*d* oxide honeycomb systems — Li_2_RhO_3_, Li_2_IrO_3_, Na_2_IrO_3_ — the smaller ligand ionic charge in the halide gives rise to significantly weaker *t*_2*g*_ − *e*_*g*_ splittings. This is apparent in Table 2 in the main text: for the *C*2/*m* crystalline structure of Cao *et al*.^28^, we compute excitation energies of only ≈1.3 eV for the lowest 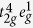 states. Even more suggestive in this regard is the energy-level diagram we compute for the *P*3_1_12 crystalline structure of ref. 29. For the latter, the sequence of Ru^3+^ 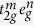 levels is shown in Table 4: it is seen that the ^6^*A*_1_ 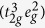 state is even lower in energy than ^4^*T*_1_ 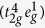. Such low-lying 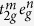 excited states may obviously play a more important role than in the oxides in intersite superexchange.

Ru 4*d*^5^ *g* factors were computed following the procedure described in ref. 35. The values provided in the main text, *g*_*ab*_ = 2.51 and *g*_*c*_ = 1.09, were obtained by including the ^2^*T*_2_ 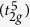, ^4^*T*_1_ 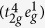, ^4^*T*_2_ 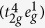, and ^6^*A*_1_ 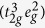 states in the spin-orbit treatment. The orbitals were optimized for an average of all these states. The strength of the coupling to external magnetic field can also be extracted from more involved calculations as described in the next subsection.

The effect of on-site 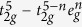 mixing on the computed *g* factors appears to be modest — by spin-orbit CASSCF calculations based on a minimal orbital active space (three Ru *t*_2*g*_ orbitals), *g*_*ab*_ = 2.63 and *g*_*c*_ = 1.03; if 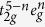 states are also considered by CASSCF (as described above), *g*_*ab*_ = 2.59 and *g*_*c*_ = 1.18.

### Intersite exchange

NN magnetic coupling constants were derived from CASSCF + MRCI spin-orbit calculations on units of two edge-sharing [RuCl_6_]^3−^ octahedra. Similar to the computations for the on-site excitations, the four octahedra adjacent to the reference [Ru_2_Cl_10_]^4−^ entity were also included in the actual (embedded) cluster. We used energy-consistent relativistic pseudopotentials along with valence basis sets of quadruple-zeta quality for the two Ru cations in the reference unit^44^. All-electron basis sets of quintuple-zeta quality were employed for the bridging ligands and triple-zeta basis functions for the remaining chlorine anions of the reference octahedra^45^. We further utilized two *f* polarization functions^44^ for each Ru ion of the central, reference unit and four *d* polarization functions^45^ at each of the two bridging ligand sites. Ru^3+^ ions of the four adjacent octahedra were modeled as closed-shell Rh^3+^ species, following a strategy similar to the calculations for the on-site 4*d*-shell transitions. The same computational scheme yields magnetic coupling constants in very good agreement with experimental estimates in CaIrO_3_^47^, Ba_2_IrO_4_^53^, and Sr_2_IrO_4_^35^,^54^.

The mapping of the *ab initio* quantum chemistry data onto the effective spin model defined by (1) implies the lowest four spin-orbit states associated with the different possible couplings of two NN 1/2 pseudospins. The other 32 spin-orbit states within the 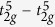 manifold^16^,^32^ involve *j*_eff_ ≈ 3/2 to *j*_eff_ ≈ 1/2 charge excitations^7^,^32^ and lie at 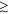 150 meV higher energy (see Tables 1 and 2 and refs 32 and 36), an energy scale much larger than the strength of intersite exchange. To derive numerical values for all effective spin interactions allowed by symmetry in (1), we additionally consider the Zeeman coupling


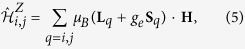


where **L**_*q*_ and **S**_*q*_ are angular-momentum and spin operators at a given Ru site while *g*_*e*_ and *μ*_*B*_ stand for the free-electron Landé factor and Bohr magneton, respectively (see also ref. 35). Each of the resulting matrix elements 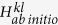 computed at the quantum chemistry level, see Table 5, is assimilated to the corresponding matrix element 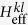 of the effective spin Hamiltonian, see Table 6. This one-to-one correspondence between *ab initio* and effective-model matrix elements enable an assessment of all coupling constants in (1).

For *C*_2*h*_ symmetry of the [Ru_2_Cl_10_] unit^28^, it is convenient to choose a reference frame with one of the axes along the Ru-Ru link. The data collected in Tables 5 and 6 are expressed by using such a coordinate system, with the *x* axis along the Ru-Ru segment and *z* perpendicular to the Ru_2_Cl_2_ plaquette. The 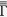 tensor reads then


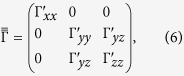


where 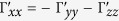 and the “prime” notation refers to this particular coordinate system. The Kitaev-like reference frame within which the data in Table 3 are expressed implies a rotation by 45° about the *z* axis^16^,^17^,^32^. The connection between the parameters of Table 3, corresponding to the Kitaev-like axes, and the “prime” quantities in Tables 5 and 6 is given by the following relations^16^,^17^,^32^:


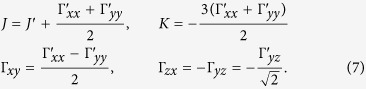


The terms Δ_*n*_ and Ω_*n*_ in Table 6 (where *n* ∈ {*y, z*}) stand for:


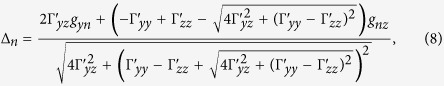



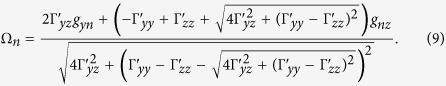


The *g* factors are here expressed in the local coordinate frame related to the Ru_2_Cl_2_ plaquette, different from the values provided in Sec. 2.

### Magnetization curves for the Kitaev-Heisenberg model

**Figure 5:**
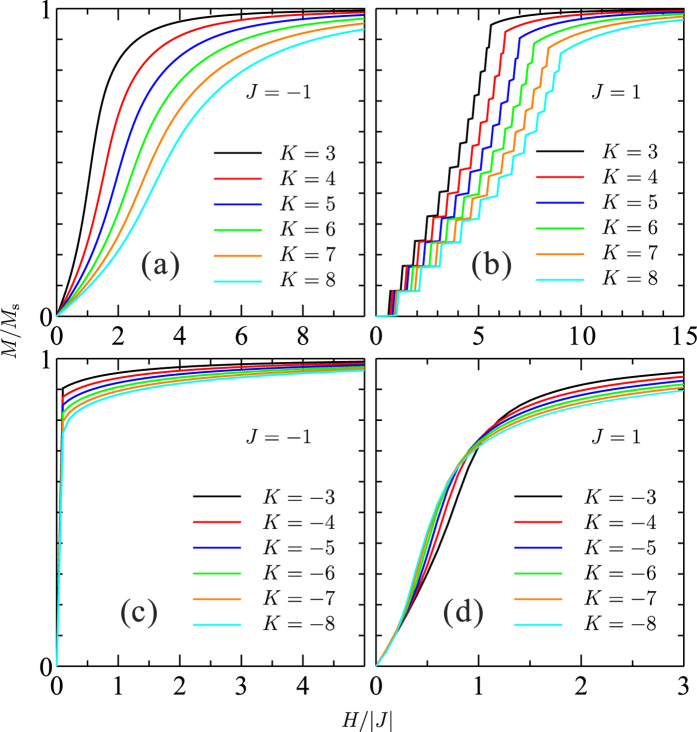
Magnetization curves of the pure Kitaev-Heisenberg model. (**a**) *J* < 0, *K* > 0. (**b**) *J* > 0, *K* > 0. (**c**) *J* < 0, *K* < 0. (**d**) *J* > 0, *K* < 0. The magnetic field is applied along the *c* direction and the saturation of the magnetization is set to be *M* = *M*_*s*_.

Magnetization curves of the pure Kitaev-Heisenberg model, calculated by ED on a 24-site cluster, are plotted in Fig. 5. The overall shapes are qualitatively well determined once the signs of *J* and *K* are fixed. For *J* > 0 and *K* > 0 [Fig. 5(b)], the magnetization increases linearly at low field and more steeply at higher field. This behavior is similar to that of the two-dimensional (2D) bipartite Heisenberg systems; the main difference is the existence of a kink near the saturation, due to local AF interactions and the mixing of different *S*^*z*^-sectors. Below the kink, the NN spin correlations remain AF. For *J* < 0 and *K* < 0 [Fig. 5(c)], the magnetization “jumps” to finite values at *H* = 0^+^ and gradually saturates with increasing field. This gradual saturation is the result of local FM interactions *S*^*x*^*S*^*x*^ and *S*^*y*^*S*^*y*^. For *J* < 0 and *K* > 0 [Fig. 5(a)], the magnetization increases linearly at low field, reflecting the AF *J*, smoothly connects to the higher-field curve and then saturates gradually with increasing field, similar to the case of *J* < 0 and *K* < 0. This qualitative behavior is basically the result of competing FM *J* and AF *K*. When *K* is small, the magnetization saturates rapidly with increasing field due to the FM *J*; as the AF *K* increases, the saturation is shifted to higher field. The shape of the magnetization curve itself is almost unchanged with changing *K* and the magnetic field can be simply rescaled by *K* · *H*. Typically, the effect of a FM *K* on the magnetization curve is small but the saturation becomes slower for larger *K*. A linear increase in weak fields and very slow saturation at higher fields was experimentally observed for *α*-RuCl_3_. Such behavior is found in the calculations only for *J* > 0 and *K* < 0 [Fig. 5(d)].

Generally, the magnetization curve of the Heisenberg model is a step function in calculations on finite-size systems, due to discrete effects. However, in the Kitaev-Heisenberg model, the total *S*^*z*^ is no longer conserved due to terms such as *S*^+^*S*^+^ and *S*^−^*S*^−^. The magnetization curve can be then a smooth function. In our results, small steps are still visible in the magnetization curve for the case of *J* > 0 and *K* > 0. There, since the Néel (or zigzag) fluctuations are strong, the mixing of different *S*^*z*^-sectors is not sufficient to mask discrete effects.

### Magnetization curves with longer-range interactions

**Figure 6:**
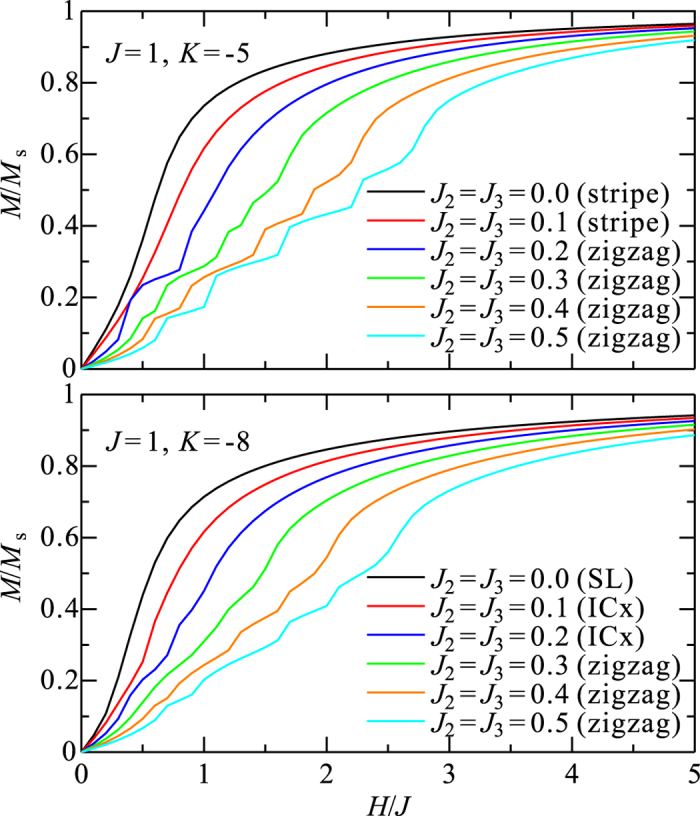
Magnetization curves of the extended Kitaev-Heisenberg model. Results with *J* = 1, *K* = −5 (top) and *J* = 1, *K* = −8 (bottom) are provided, for several values *J*_2_ = *J*_3_. For each set *J*_2_ = *J*_3_, the dominant state at *H* = 0 is indicated within parentheses. The magnetic field is applied along the *c* direction.

We find that *J* > 0 and *K* < 0 values are required to reproduce the experimental magnetization curves. Looking in more detail to the dependence on longer-range interactions *J*_2_ and *J*_3_ is also instructive. Magnetization curves at *J* = 1, *K* = −5 and *J* = 1, *K* = −8 are shown in Fig. 6 for several *J*_2_ = *J*_3_ values. The effect of longer-range interactions seems to be even quantitatively similar for the two different *K* values. As long as *J*_2_ and *J*_3_ are much smaller than |*J*| (*J*_2_, *J*_3_ < 0.2 |*J*|), the saturation is simply shifted to higher field but the overall shape of the magnetization curve is conserved. On the other hand, for *J*_2_, *J*_3_ > 0.3 |*J*|, the overall shape changes somewhat, approaching that for the case of *J* > 0 and *K* > 0. We thus infer that *J*_2_ and *J*_3_ must be smaller than 0.3 |*J*| to reproduce the experimental magnetization curves. Only results for the case of *J*_2_ = *J*_3_ are shown here for simplicity, since we find that *J*_2_ and *J*_3_ have similar effect on the magnetization curves and affect those almost independently.

### Spin-spin correlations in the spin-liquid phase

**Figure 7:**
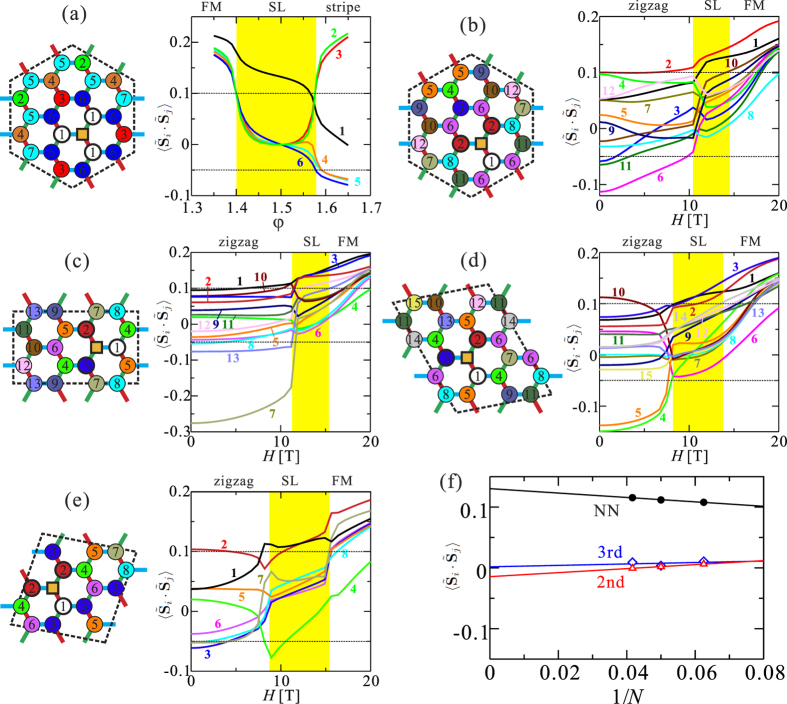
Spin-spin correlation functions 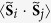. (**a**) ED results for the NN Kitaev-Heisenberg model. (**b**–**e**) ED results for our extended spin model, using MRCI *g* factors and NN couplings plus *J*_2_ = *J*_3_ = 0.25 meV. The clusters used in the ED calculations are also sketched. The reference site is indicated by a square and the numbers labeling various other sites are in direct correspondence with the numbered curves in the plots of 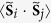. Yellow windows indicate the Kitaev SL region. (**f** ) Finite-size scaling analysis for the NN, second- and third-neighbor spin-spin correlation functions at *H* = 13.2 T.

**Figure 8:**
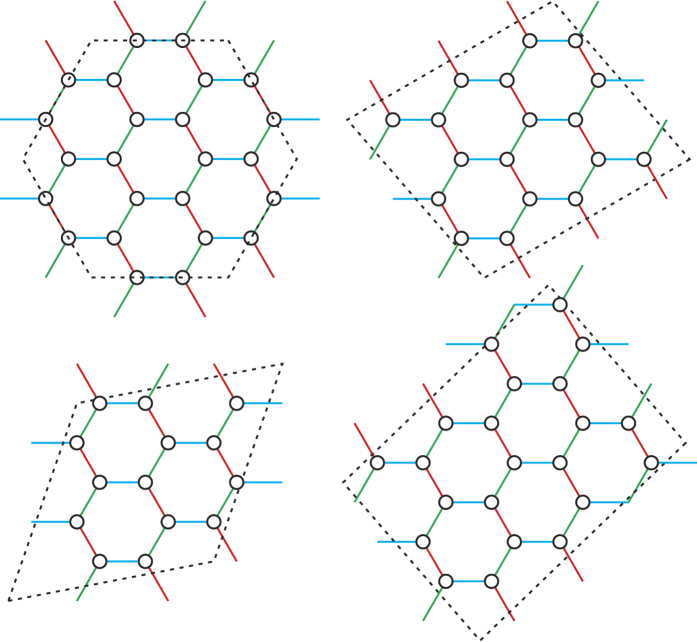
PBC clusters geometrically inconsistent with zigzag order.

To describe in more detail the Kitaev SL phase in the intermediate-field region, we calculated the field-dependent spin-spin correlation functions 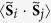 and compared them to those of the zero-field Kitaev SL phase of the 2D Kitaev-Heisenberg model on the honeycomb lattice^8^. The NN interactions of the Kitaev-Heisenberg model can be written as


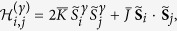


where *γ*(=*x, y, z*) labels the three distinct types of NN bonds in the “regular” honeycomb plane. Following the notation of ref. 8, we define the effective parameter 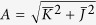 and an angle *φ* via 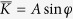 and 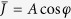. In Fig. 7(a), spin-spin correlations near the FM Kitaev limit (*φ* = 1.5) of the Kitaev-Heisenberg model are plotted, for a 24-site cluster with PBC’s. The Kitaev SL state is characterized by a rapid decay of the spin-spin correlations: in the Kitaev limit, only the NN correlations are finite and longer-range ones are zero; that is faithfully reproduced by the 24-site calculations. Even away from the Kitaev limit, the longer-range (not NN) spin-spin correlations fall within a narrow range 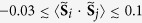 in the Kitaev SL phase (1.40 < *φ* < 1.58). As seen in Fig. 7(b), using the same 24-site cluster, our field-induced SL state exhibits similar features; the values of longer-range correlations are distributed within a narrow range 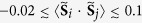 in the SL phase (10.8 T < *H* < 14.2 T). In other words, a rapid decay of the spin-spin correlations is seen in our field-induced SL state, at the same level as in the FM Kitaev SL phase of the 2D Kitaev-Heisenberg model. We also found that the zigzag-SL level crossing and the associated rapid decay of the spin-spin correlations occur for any cluster which can stabilize a zigzag-ordered ground state at low or no field [see Fig. 7(c–e)]. On the other hand, there is no level crossing for clusters geometrically inconsistent with zigzag order, e.g., the clusters depicted in Fig. 8. Finite-size scaling analysis of the NN, second-neighbor and third-neighbor spin-spin correlation functions within the SL phase (*H* = 13.2 T) is shown in Fig. 7(f). The rather small dependence on cluster size is a natural consequence of having no finite-size effects in the Kitaev limit.

## Additional Information

**How to cite this article**: Yadav, R. *et al*. Kitaev exchange and field-induced quantum spin-liquid states in honeycomb *α*-RuCl_3_. *Sci. Rep.* **6**, 37925; doi: 10.1038/srep37925 (2016).

**Publisher's note:** Springer Nature remains neutral with regard to jurisdictional claims in published maps and institutional affiliations.

## Figures and Tables

**Table 1:** Ru^3+^ 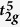 wave functions (hole picture) and relative energies (meV).

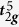 states (CASSCF)	Relative energies	Wave-function composition (normalized weights, %)
Sans SOC:		
|*ϕ*_1_〉	0	99.75|*α*〉 + 0.25|*β*〉
|*ϕ*_2_〉	69	100|*γ*〉
|*ϕ*_3_〉	72	0.25|*α*〉 + 99.75|*β*〉
With SOC:		
|*ψ*_1_〉	0	55|*ϕ*_1_, ↓〉 + 23|*ϕ*_2_, ↑〉 + 22|*ϕ*_3_, ↑〉
|*ψ*_2_〉	157	45|*ϕ*_1_, ↑〉 + 29|*ϕ*_2_, ↓〉 + 26|*ϕ*_3_, ↓〉
|*ψ*_3_〉	198	48|*ϕ*_2_, ↑〉 + 52|*ϕ*_3_, ↓〉

CASSCF results sans and with SOC for the crystal structure of ref. 28. Only the 4*d t*_2*g*_ orbitals were active in CASSCF; by subsequent MRCI, the energies change to 0, 66, 73 sans SOC and to 0, 162, 201 with SOC included. Only one component of the Kramers’ doublet is shown for each CASSCF + SOC relative energy. |*α*〉 corresponds to the *a*_1*g*_ function while |*β*〉, |*γ*〉 are 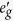 components^27^.

**Table 2:** Ru^3+^ 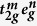 splittings (eV), with all five 4*d* orbitals active in CASSCF.

Ru^3+^ 4*d*^5^ splittings	CASSCF	CASSCF + SOC	MRCI	MRCI + SOC
^2^*T*_2_ 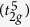	0	0	0	0
	0.066	0.193	0.067	0.195
	0.069	0.232	0.071	0.234
^4^*T*_1_ 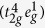	1.08	1.25	1.28	1.33
	1.12	|	1.30	|
	1.13	1.37	1.31	1.48
^4^*T*_2_ 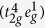	1.76	1.90	1.97	2.09
	1.81	|	2.01	|
	1.83	1.98	2.03	2.17
^6^*A*_1_ 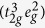	1.01	1.09 (×6)	1.51	1.74 (×6)

Except lowest line, each spin-orbit relative-energy entry implies a Kramers doublet. Just the lowest and highest components are depicted for each group of 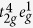 spin-orbit states. Only the *T* and *A* states shown in the table entered the spin-orbit calculations.

**Table 3:** MRCI NN magnetic couplings (meV).

Structure	∠Ru-Cl-Ru	*K* = Γ_*zz*_	*J*	Γ_*xy*_	Γ_*zx*_ = −Γ_*yz*_
*C*2/*m*^28^	94°	−5.6	1.2	−1.2	−0.7
*C*2/*m*^30^					
Link 1 (×2)	94°	−5.3	1.2	−1.1	−0.7
Link 2 (×1)	93°	−4.8	−0.3	−1.5	−0.7
*P*3_1_12^29^	89°	−1.2	−0.5	−1.0	−0.4

Three different crystal structures proposed for *α*-RuCl_3_ were analyzed. For the structure determined in ref. 30, the two crystallographically different NN Ru-Ru links are also different magnetically.

**Table 4:** Ru^3+^ 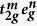 splittings (eV) in the crystalline structure of ref. 29.

Ru^3+^ 4*d*^5^ splittings	CASSCF	CASSCF + SOC	MRCI	MRCI + SOC
^2^*T*_2_ 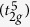	0	0	0	0
	0.04	0.16	0.05	0.19
	0.05	0.16	0.06	0.23
^6^*A*_1_ 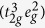	0.07	0.21 (×6)	0.92	0.92 (×6)
^4^*T*_1_ 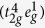	0.62	0.78	0.94	1.10
	0.66	|	0.97	|
	0.66	0.85	0.98	1.23
^4^*T*_2_ 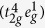	1.27	1.42	1.52	1.65
	1.33	|	1.56	|
	1.38	1.55	1.63	1.77

Except the 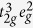 states, each spin-orbit relative-energy entry implies a Kramers doublet. Just the lowest and highest components are depicted for each group of 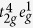 spin-orbit states. Only the *T* and *A* states shown in the table entered the spin-orbit calculations.

**Table 5:** Matrix elements of the *ab initio* model Hamiltonian (meV), as obtained by spin-orbit MRCI.

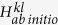	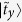	|*t*_*x*_〉	|*s*〉	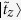
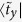	0	0.804*iμ*_*B*_*H*_*y*_ + 2.720*iμ*_*B*_*H*_*z*_	0	−1.826*iμ*_*B*_*H*_*x*_
〈*t*_*x*_|	−0.804*iμ*_*B*_*H*_*y*_ − 2.720*iμ*_*B*_*H*_*z*_	1.189	0	−1.130*iμ*_*B*_*H*_*y*_ − 0.280*iμ*_*B*_*H*_*z*_
〈*s*|	0	0	2.187	0
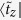	1.826*iμ*_*B*_*H*_*x*_	1.130*iμ*_*B*_*H*_*y*_ + 0.280*iμ*_*B*_*H*_*z*_	0	3.475

The two-site singlet and (split) triplet states are labeled |*s*〉 and 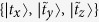, respectively. 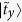 and 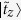 are admixtures of ‘pure’ |1, −1〉 and |1, 0〉 spin functions.

**Table 6:** Matrix form of the effective spin Hamiltonian in the basis of zero-field eigenstates.

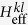	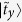	|*t*_*x*_〉	|*s*〉	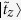
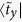	0	*iH*_*y*_Δ_*y*_ + *iH*_*z*_Δ_*z*_	0	*ig*_*xx*_*H*_*x*_
〈*t*_*x*_|	−*iH*_*y*_Δ_*y*_ − *iH*_*z*_Δ_*z*_	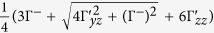	0	*iH*_*y*_Ω_*y*_ + *iH*_*z*_Ω_*z*_
〈*s*|	0	0	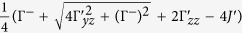	0
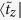	−*ig*_*xx*_*H*_*x*_	−*iH*_*y*_Ω_*y*_ − *iH*_*z*_Ω_*z*_	0	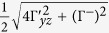

Γ^−^ stands for 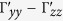; expressions for the Δ and Ω terms are provided in text.
